# Durable Complete Response to Alectinib in a Lung Adenocarcinoma Patient With Brain Metastases and Low-Abundance *EML4-ALK* Variant in Liquid Biopsy: A Case Report

**DOI:** 10.3389/fonc.2020.01259

**Published:** 2020-07-31

**Authors:** Yingying Zhu, Ran Jia, Yang W. Shao, Liuqing Zhu, Qiuxiang Ou, Man Yu, Xue Wu, Yanbei Zhang

**Affiliations:** ^1^Department of Geriatric Respiratory and Critical Care, Institute of Respiratory Diseases, The First Affiliated Hospital of Anhui Medical University, Hefei, China; ^2^Department of Hepatobiliary Surgery, The First Affiliated Hospital of Anhui Medical University, Hefei, China; ^3^Medical Department, Nanjing Geneseeq Technology Inc., Nanjing, China; ^4^Translational Medicine Research Institute, Geneseeq Technology Inc., Toronto, ON, Canada

**Keywords:** lung adenocarcinoma, *ALK* rearrangement, alectinib, liquid biopsy, brain metastases

## Abstract

*EML4-ALK* fusions are targetable oncogenic drivers in a subset of advanced non-small cell lung cancer (NSCLC) patients that can benefit from selected ALK inhibitors. Precise detection of *ALK* fusions may yield critical information for selection of appropriate therapy and hence improve patient survival. Analysis of circulating tumor DNA (ctDNA) in liquid biopsies using next generation sequencing (NGS) prior to or during treatment hold great promise for disease monitoring and treatment guidance of various cancers including NSCLC. Herein, we report a case of a 21-year-old advanced lung adenocarcinoma patient with a low abundance (0.03%) of *EML4-ALK* rearrangement identified in plasma ctDNA upon progression on two lines of chemotherapy that demonstrated long-term complete response to alectinib (>13 months) including metastatic brain tumors. Patient's clinical and pathologic characteristics, computerized tomography (CT) scans and brain magnetic resonance imaging (MRI) were reviewed retrospectively. Taken together, our report not only reinforces the translational utility of NGS-based genomic sequencing of liquid biopsy in guiding clinical practice, but also highlights the superior efficacy of alectinib than chemotherapy in *ALK*+ NSCLC with brain metastases, albeit at a low variant allele abundance.

## Introduction

Aberrant *ALK* rearrangements have been recognized as central oncogenic drivers for many solid malignancies. *EML4-ALK* fusions occur in ~2–7% of advanced non-small cell lung cancer (NSCLC) patients, and are more frequently detected in lung adenocarcinoma as well as in never- or light- smokers or young adults (1). Despite of high overall response rates (ORR) with the first-generation ALK inhibitor crizotinib (2), drug resistance inevitably develops with the central nervous system (CNS) as the most common site of progressive disease in nearly 70% of *ALK*+ patients undergoing crizotinib treatment. Approximately 15–35% of *ALK*+ NSCLC patients manifest with CNS metastases at initial diagnosis (3), dramatically impacting patient prognosis and quality of life. Previous studies have provided convincing evidence for the superior potency and improved tolerability of alectinib over crizotinib in *ALK*+ patients with baseline brain metastases or leptomeningeal disease (4, 5), which underlines the potential efficacy of alectinib in treating *ALK*-driven NSCLC, particularly in the management of those harboring CNS metastases. Analysis of circulating tumor DNA (ctDNA) in liquid biopsies using next generation sequencing (NGS) provide a non-invasive approach to tumor molecular profiling and is increasingly utilized to screen presence of disease, guide therapy selection, and evaluate treatment response (6). However, adequate assessment of low-abundance ctDNA alterations and their translational significance may be challenging under some circumstances. In this case study, we report a lung adenocarcinoma patient with brain metastases whose disease progressed upon chemotherapy but responded completely to alectinib with detection of a low-abundance *EML4-ALK* fusion in plasma ctDNA.

## Case Presentation

**Figure 1:**
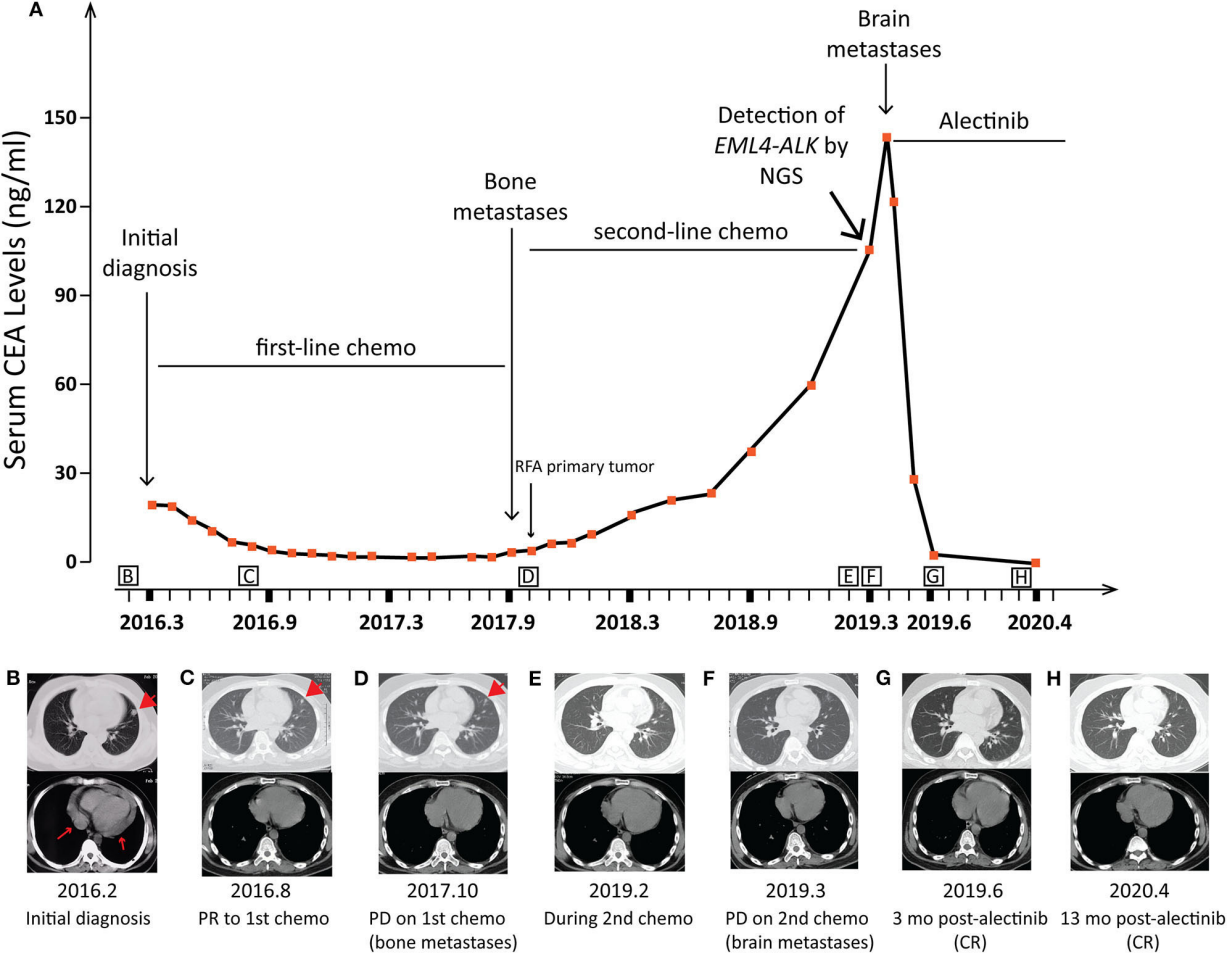
Schematics showing the patient's treatment history. **(A)** A diagram showing the chronological changes of therapeutic regimens and serum CEA levels during the entire course of treatment. RFA, radiofrequency ablation. **(B–H)** The patient's chest computed tomography (CT) scans at different clinical time points as shown. Arrowheads: location of the primary lung lesion in different scans. Arrows: paricardial effusion at initial diagnosis. PR, partial response; PD, progressed disease.

A 21-year-old Chinese male with neither personal smoking history nor family medical history was diagnosed with stage IV lung adenocarcinoma with multiple metastases in cervical, hilar and mediastinal lymph nodes, and pericardial effusion in February 2016 (Figures 1A,B). Cervical lymph node biopsy was performed but not the primary lung tumor due to a number of reasons including the tumor size (1.5 cm by 1.5 cm), its proximity to the heart, and a large accumulation of pericardial effusion present at diagnosis. The baseline lymph node biopsy specimen, pericardial effusion ctDNA, and plasma ctDNA samples were immediately subject to comprehensive genomic profiling using next generation sequencing (NGS) by targeting 382 cancer-relevant genes, but no actionable driver mutations were detected in any sample (Table 1). FISH or IHC against biomarkers including ALK was not adopted for routine clinical diagnosis at our institution in 2016. The patient soon received the first-line chemotherapy of six cycles of pemetrexed (500 mg/m^2^) and cisplatin (75 mg/m^2^) and achieved a partial response (PR) according to the RECIST guideline version 1.1(7) (Figure 1C). No mutation was detected in the post-chemo plasma ctDNA sample (August 2016) using NGS by the same targeted gene panel (Table 1). A maintenance chemotherapy continued with ten cycles of pemetrexed (500 mg/m^2^). The patient demonstrated a progression-free survival (PFS) of about 20 months in total during the course of first-line chemotherapy until the disease progressed with the occurrence of bone metastases in October 2017, although the primary lung lesion remained stable (Figure 1D).

**Table 1:** Genetic alterations identified by NGS in the patient's FFPE and liquid biopsy samples.

**Time points (in Figure 1A)**	**Sample type**	**Gene**	**AA change**	**MAF%**
At diagnosis (2016.2)	FFPE (cervical lymph node)	*ARID1A*	p.G87X	37.00
		*STAT3*	p.E166Q	2.00
		*TP53*	p.T155N	61.00
	Plasma ctDNA	*-*		
PR to 1st-line chemotherapy (2016.3)	Pericardial effusion ctDNA	*BRCA1*	p.Q1240X	2.00
		*ARID1A*	p.Q2100X	4.00
		*CTNNB1*	p.D32H	4.00
		*TP53*	p.T155N	7.00
PR to 1st-line chemotherapy (2016.8)	Plasma ctDNA	*-*		
PD on 2nd-line chemotherapy (2019.3)	Plasma ctDNA	*ARID1A*	p.Q2100X	0.20
		*TP53*	p.T23N	0.40
		*EML4-ALK (E6:A20)*		0.03

*PR, partial response; PD, progressive disease; ctDNA, circulating tumor DNA; AA, amino acid; MAF, mutant allele frequency; “-,” no somatic alterations detected*.

**Figure 2:**
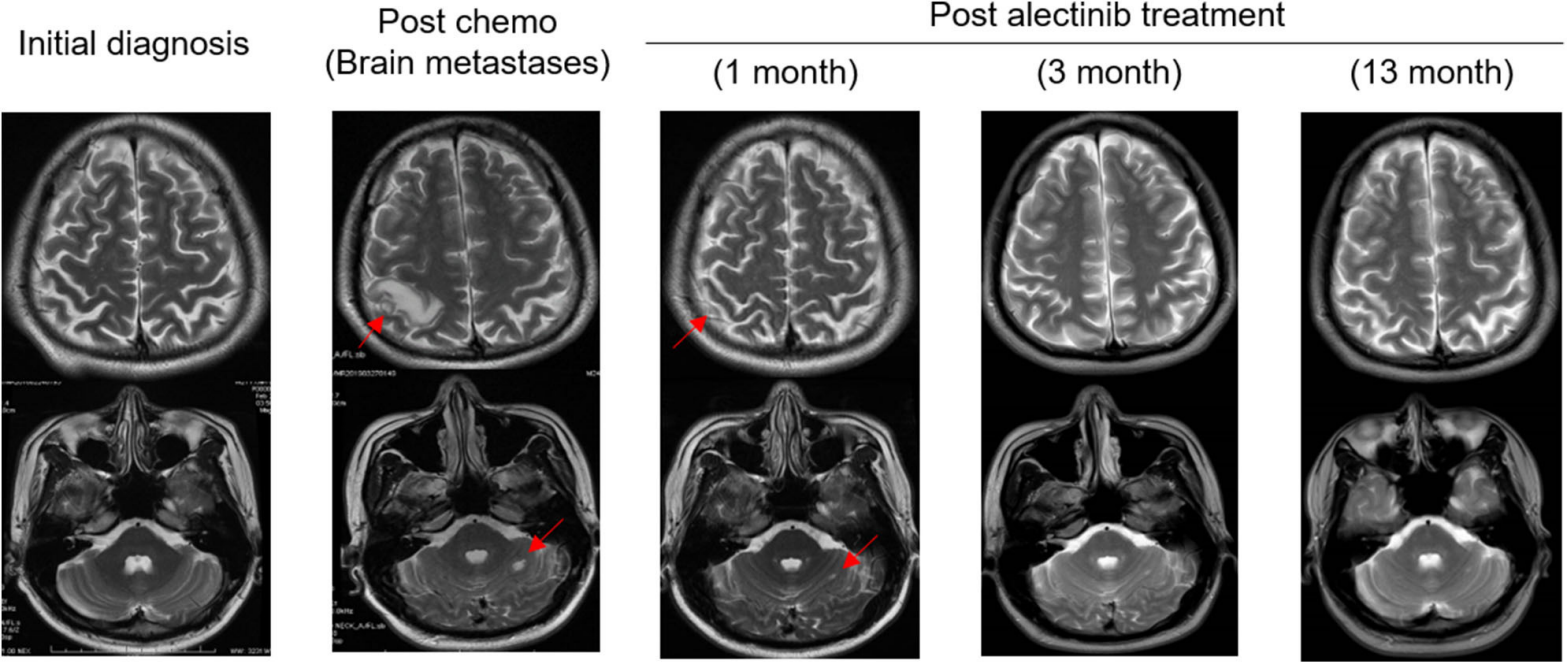
Serial MRI scans showing patient's metastatic brain tumors responded completely to alectinib therapy. The patient achieved partial response (PR) 1 month after the initiation of alectinib therapy, and the lesions disappeared (CR) completely at the 3- and 13- month post-alectinib treatment time points. Arrows: metastatic brain tumors. CR, complete response.

The patient was then treated with radiofrequency (RF) ablation for the primary lung lesion in October 2017, followed by two cycles of second-line pemetrexed (500 mg/m^2^) and carboplatin (6 mg/ml/min). In January 2018, the patient was switched to single-agent pemetrexed (5 cycles, 500 mg/m^2^) due to severe allergic reactions to carboplatin. The primary lung tumor demonstrated durable complete response to second-line chemotherapy (Figure 1E). However, serum CEA levels steadily increased and reached 105.4 μg/L by February 2019 (Figure 1A). Remarkably, an *EML4-ALK* fusion variant (*E6:A20*) was detected at a low allele frequency (AF) of 0.03% in plasma ctDNA using the same targeted NGS panel (Table 1). However, it could not be validated by IHC or FISH due to an insufficient quantity of primary lesion for biopsy. Considering that there were few or no other treatment options, despite a low abundance of *EML4-ALK* fusion, we still determined to treat this patient with alectinib (600 mg twice daily) in March 2019 upon the diagnosis of multiple brain metastases by brain magnetic resonance imaging (MRI; Figures 1F, 2). CEA levels declined markedly (Figure 1A) following the treatment and a significant reduction in the size of brain lesions was also observed (Figure 2). After 3 months, metastatic brain tumors disappeared completely, while the primary lung lesion remained under control (Figure 1G) and CEA levels dropped to 2.2 μg/L (Figure 1A). The patient remained relapse-free during the entire follow-up period of about 13 months up till April 2020 (Figures 1H, 2).

## Discussion

Here, we describe an advanced lung adenocarcinoma case with a low-abundance *EML4-ALK* fusion detected in plasma ctDNA. This patient had a progression of disease after two lines of chemotherapy but displayed a durable complete response to alectinib including brain metastases. In accordance with the results of the ALEX phase three trial (5), alectinib showed potent systemic and CNS efficacy and low toxicity in the patients carrying a low frequency of *EML4-ALK* fusion, further consolidating alectinib as the standard of care for untreated, advanced *ALK*+ NSCLC, irrespective from the presence or absence of baseline CNS metastases.

We acknowledge that the sensitivity of fusion detection in cell-free DNA (cfDNA) was reported to be lower than that for mutations or indels (8), and differences in fusion detection were also noted between different cfDNA NGS assays (9). However, cfDNA should be considered as a rule in vs. a rule out test even if the AF is lower than the reportable threshold of the cfDNA assay. Furthermore, it has been largely debated on TKI efficacy irrespective of mutation AF in advanced NSCLC including *EGFR*-mutant tumors. Given that a high abundance of *EGFR* activating mutation was reported to be significantly associated with better objective response to EGFR TKIs and greater PFS benefits (10, 11), a treatment regimen of chemotherapy in combination with TKIs may be considered for advanced NSCLC with *EGFR* activating mutations of low AF. It is also worth noting that this *EML4-ALK* aberration was not detected at diagnosis or during first-line chemotherapy by the same NGS-based mutation panel under the same detection threshold. This tumor-plasma discordance may be partly explained by a high degree of tumor heterogeneity of the patient, highlighting the importance of intra-patient tumor heterogeneity as previously reported in *EGFR*-mutant NSCLC (12–14). Together, these data underscored the translational utility of NGS-based genomic sequencing of liquid biopsy in guiding clinical practice, which allows a more comprehensive analysis of tumor heterogeneity.

Although alectinib has demonstrated potent antitumor activity against *ALK*-rearranged NSCLC, the disease inevitably relapses in the clinic mainly owing to acquired therapy resistance mediated by multiple mechanisms. A number of ALK inhibitors including brigatinib (15) and lorlatinib (16) have been documented to have highly selective activity against *ALK* mutants resistant to first- and second- generation ALK-TKIs. In particular, lorlatinib was reported to be very active against almost all *ALK* mutants, including the G1202R variant. More importantly, lorlatinib displayed a strong brain-penetrant property and anticancer potency toward intracranial metastatic tumors in a phase 1, dose-escalation trial of advanced *ALK*- or *ROS1*-positive NSCLC patients, most of whom had CNS metastases (17). Late phases of clinical trials of third generation ALK inhibitors are currently undergoing, which may open up a new avenue for patients who develop brain metastases after the acquisition of resistance mechanisms to currently available ALK-TKIs.

In conclusion, this case report emphasizes the importance of NGS-based genomic sequencing of liquid biopsy in disease monitoring and therapy guidance and highlights the superior efficacy of alectinib than chemotherapy in primary treatment of *ALK*+ NSCLC patients with CNS metastases, including those with low-abundance *ALK* rearrangements.

## Laboratory Investigations and Diagnostic Tests

Comprehensive genomic profiling was performed using next generation sequencing by targeting 382 cancer-relevant genes in a Clinical Laboratory Improvement Amendments-certified, College of American Pathologists-accredited laboratory (Nanjing Geneseeq Technology, Nanjing, Jiangsu Province, China). In brief, genomic DNA were extracted from cervical lymph node biopsy specimen using the DNeasy Blood & Tissue kit (Qiagen) according to the manufacturer's protocols. Cell-free (cfDNA) from pericardial effusion or plasma samples was extracted using the QIAamp Circulating Nucleic Acid kit (Qiagen). Approximately 200 ng of cfDNA was used for subsequent library preparation using the KAPA Hyper Prep kit (KAPA Biosystems) according to manufacturer's suggestions for different sample types. Sequencing library preparation, targeted gene enrichment, and sequencing data processing were carried out following the methods as previously described (18).

## Clinical Practice Points

- An advanced lung adenocarcinoma patient with a low abundance of *EML4-ALK* fusion demonstrated a durable complete response to alectinib.
- Analysis of plasma ctDNA changes using NGS-based liquid biopsy assays holds great promise for tracing disease progression or recurrence and guiding treatment decision-making.

## Data Availability Statement

The datasets generated for this study are available on request to the corresponding author.

## Ethics Statement

The studies involving human participants were reviewed and approved by the First Affiliated Hospital of Anhui Medical University, Hefei, Anhui, China. The patients/participants provided their written informed consent to participate in this study. Written informed consent was obtained from the patient for publication of this case report and any accompanying images.

## Author Contributions

YZhu and RJ conducted data curation and project management. YS, LZ, QO, and XW reviewed and analyzed data. YZha designed the concept and methodology and supervised the entire study. QO, MY, and YZha wrote the manuscript and all authors read and approved the final manuscript. All authors contributed to the article and approved the submitted version.

## Conflict of Interest

YS and LZ are the employees of Nanjing Geneseeq Technology Inc., China. QO, MY, and XW are the employees of Geneseeq Technology Inc., Canada. The remaining authors declare that the research was conducted in the absence of any commercial or financial relationships that could be construed as a potential conflict of interest.
